# Provider perspectives on contraceptive service delivery: findings from a qualitative study in Johannesburg, South Africa

**DOI:** 10.1186/s12913-020-4900-9

**Published:** 2020-02-21

**Authors:** Naomi Lince-Deroche, Cheryl Hendrickson, Aneesa Moolla, Sharon Kgowedi, Masangu Mulongo

**Affiliations:** 1 Department of Internal Medicine, Health Economics and Epidemiology Research Office, School of Clinical Medicine, Faculty of Health Sciences, University of the Witwatersrand, Johannesburg, South Africa; 2 Right to Care, Johannesburg, South Africa

**Keywords:** Contraception, HIV, Family planning, Healthcare providers, Implants, Integration

## Abstract

**Background:**

Healthcare providers’ skills and attitudes are both barriers and facilitators of contraceptive uptake. In South Africa, migration of healthcare workers and the demands of the HIV epidemic have also contributed to inequitable access to sexual and reproductive health (SRH) care. Yet, the country has committed to achieving universal access to SRH services. We explored healthcare provider’s opinions and attitudes on provision of contraceptive services in public facilities, their personal use of methods, and their thoughts on the recent integration of new contraceptive methods in their facilities.

**Methods:**

We conducted a phenomenological, qualitative study in 2017 at an outpatient, public HIV treatment clinic and two primary healthcare clinics (PHCs) in Johannesburg, South Africa. We purposively selected providers who had worked at the facilities for at least six months and were seeing patients for HIV or SRH services. Trained study staff conducted semi-structured interviews. We conducted descriptive analyses for quantitative data, and used an iterative, thematic analysis approach for open-ended responses.

**Results:**

We interviewed 14 healthcare providers (HIV clinic - 5; PHCs - 9). One respondent was a man; all were nurses. All respondents reported having ever personally used a contraceptive method; half (7/14) were currently using a method. Responses on service provision were conflicting. Respondents felt that their clinics currently met the contraceptive needs of their female patients through on-site services or referrals. However, they noted that staff shortages, lack of training, and a limited contraceptive offering meant that women did not always get the counselling or method they wanted. Respondents noted that the ‘best’ contraceptive methods for women were those that fit with a woman’s lifestyle and medical needs; however, providers also felt strongly that injectables were best for all women. Recent introduction of the implant at one PHC and injectable contraceptives at the HIV clinic was not overly challenging, though there were concerns about staffing and demand creation for the new methods.

**Conclusions:**

Respondents’ conflicting responses revealed challenges with current service delivery, particularly contraceptive counselling. Addressing staff workloads and providing refresher training on contraception would contribute to increased contraceptive service capacity and quality in this setting.

## Background

Providers’ skills and attitudes towards provision of contraceptive methods and other sexual and reproductive health (SRH) services have repeatedly been shown to serve as both barriers and facilitators of service uptake [1–5]. In the early 1990s, Bruce et al. posited a framework for assessing the quality of family planning programs that highlighted the central role of healthcare providers as counsellors and guides to women seeking services [6, 7]. Unfortunately, healthcare providers—for all health services—are in short supply globally, with Southeast Asia and Africa most affected [8]. In addition, significant international and internal migration of healthcare workers has resulted in inequitable access to care in many areas and pressure to expand scopes of practice [9, 10].

South Africa has been impacted by both outward migration of locally trained healthcare workers and increased workloads due to the country’s HIV epidemic [11], which is one of the world’s worst in terms of the number of people requiring care and treatment [12]. The country falls behind similarly developed countries in terms of the number of healthcare providers per capita [13, 14]. In 2013, South Africa had 0.74 physicians per 1000 people; whereas Brazil had 1.85 and Mexico had 2.07 [14]. Despite a focus on integration of HIV and other primary care services in many policy documents [15–20], there are numerous accounts of poor service quality or a lack of certain services altogether in some areas [21].

In spite of these challenges, South Africa has committed to meeting the Sustainable Development Goals broadly, including achieving universal access to SRH services by 2030 [22]. Contraceptive prevalence in South Africa was 54% among married women and 64% among sexually active women in 2016 [23]. This is high when compared to an average of 22.9% among married women in Sub-Saharan Africa as a region between 2005 and 2015 [14]. The South African National Department of Health has established goals for increasing contraceptive uptake in South Africa; an updated contraceptive policy, launched in 2013, introduced the sub-dermal contraceptive implant (the implant) and highlighted the importance of increasing access to long-acting reversible contraception [18]. However, there are still many opportunities for improvement of contraceptive services, including addressing the role of healthcare workers [24]. In particular, stigma directed toward young women trying to obtain contraception in health facilities remains a problem [24, 25]. Also, the country’s high contraceptive prevalence masks problems with consistent use [26, 27], and although in principle a broad range of methods should be available freely in public health facilities [18], most contraceptive users rely on injectable methods or male condoms [23]. Further, there were several documented challenges regarding introduction of the implant [21], including confusion about whether implants can be used by women taking antiretrovirals (ARVs) and certain other medications.

In this study, we explored healthcare provider’s opinions and attitudes on provision of contraceptive services in public sector facilities, including their thoughts on what methods are best for women, and knowledge of which methods are appropriate for HIV-positive women taking ARVs. Because health care providers’ personal use of methods has been shown to impact on their contraceptive counselling behaviour [28], we also asked about prior and current use of contraception. Finally, we explored their thoughts on the recent integration of new methods in their facilities and elicited their recommendations for addressing South Africa’s goal of universal access to SRH.

## Methods

We conducted a phenomenological, qualitative study with healthcare providers at three public healthcare facilities in Johannesburg, South Africa from March to May 2017. The facilities were purposively chosen due to offering both HIV and SRH services. One facility was an outpatient, public HIV treatment clinic, located within a large tertiary hospital. The other two facilities were public primary healthcare clinics (PHCs) that offered a range of contraceptives. The implant had been introduced at one of the PHCs in 2014. While injectables contraceptives were introduced at the HIV clinic in 2016.

We used purposive sampling to approach healthcare providers at each facility. We obtained a comprehensive list of all clinical staff members and their position titles from the facility managers, and selected possible candidates with an aim towards having representation of the various provider types (e.g. doctor, primary healthcare nurse, professional nurse, enrolled nurse, etc.). Providers were eligible for inclusion if they could speak English, had been working at the facility for at least six months and were seeing patients for HIV, prevention of mother-to-child transmission of HIV, or SRH services at the time of recruitment. (All health care workers in South Africa are trained using English as the language of instruction, so we anticipated that no one would be excluded because of the language criterion.)

Potentially eligible healthcare providers were approached either in-person or by telephone to assess their interest and availability to participate in the study. If interested and willing, trained interviewers scheduled a time to meet with the provider in-person, to formally assess study eligibility, obtain written informed consent, and administer the semi-structured interview guide, which was created for this study. The interviews were conducted in English and included basic demographics, prior and current use of contraceptives, previous contraceptive training and experience, perceptions of the appropriateness of contraceptive methods for women, and thoughts on how South Africa might improve access to contraceptive services nationally. (See Additional file 1 for further detail.)

De-identified interview data were entered into REDCap [29]. Using SAS v9.3 (SAS Institute Inc., Cary, NC, USA), we calculated frequencies for categorical data, based on non-missing responses, and present these results by facility type. Following STROBE guidelines for observational studies, we present the descriptive results without statistical testing [30]. We used NVivo (V10; QSR International Pty Ltd.) to analyse thematically the responses to open-ended questions. We also consulted the COREQ checklists for presentation of qualitative research in preparation of this manuscript [31].

## Results

### Respondent characteristics

Across the three clinics, 18 healthcare providers were approached for inclusion in the study. Four declined to participate (one did not feel comfortable being interviewed; one said she did not have time available; and two did not give reasons for their refusal). Ultimately, we interviewed 14 healthcare providers: five at the HIV clinic and nine across the two PHCs, representing 25 and 64% of the clinical healthcare providers at these respective sites (Table 1). All identified as Black racially. Only one respondent was a man. Most respondents were nurses (including the one man) because doctors were not permanently based at the PHCs. All had contraceptive training as part of their original studies, and all had experience providing contraceptive services professionally (data not shown).

**Table 1:** Healthcare worker demographics and SRH training and experience in three public health facilities Johannesburg (n (%))

	HIV clinic (*n* = 5)	PHC clinics (*n* = 9)	Total (*n* = 14)
Current job title			
Staff/enrolled nurse	2 (40)	1 (11)	3 (21)
Professional nurse	1 (20)	5 (56)	6 (43)
Primary healthcare nurse	0 (0)	1 (11)	1 (7)
Manager/nurse	1 (20)	2 (22)	3 (21)
Doctor/medical officer	1 (20)	0 (0)	1 (7)
Years of experience in current position			
< 5 years	0 (0)	2 (22)	2 (14)
≥ 5 years	5 (100)	7 (78)	12 (86)
Has personally ever used … for contraception			
Tubal ligation/hysterectomy	1 (20)	2 (22)	3 (21)
Male vasectomy	0 (0)	0 (0)	0 (0)
Intrauterine contraceptive device	2 (40)	1 (11)	3 (21)
Contraceptive implant	0 (0)	2 (22)	2 (14)
Injectable contraception	4 (80)	7 (78)	11 (79)
Contraceptive pills	3 (60)	6 (67)	9 (64)
Emergency contraception	1 (20)	3 (33)	4 (29)
Male condoms	4 (80)	5 (56)	9 (64)
Female condoms	2 (40)	1 (11)	3 (21)
Currently personally uses … for contraception			
Tubal ligation/hysterectomy	1 (20)	2 (22)	3 (21)
Male vasectomy	0 (0)	0 (0)	0 (0)
Intrauterine contraceptive device	0 (0)	0 (0)	0 (0)
Contraceptive implant	0 (0)	1 (11)	1 (7)
Injectable contraception	1 (20)	1 (11)	2 (14)
Contraceptive pills	0 (0)	0 (0)	0 (0)
Emergency contraception	0 (0)	0 (0)	0 (0)
Male condoms	0 (0)	3 (33)	3 (21)
Female condoms	0 (0)	0 (0)	0 (0)

*PHC* Primary healthcare clinics, *FP* family planning/contraception, *SRH* sexual and reproductive health

None of the facilities offered the full contraceptive method mix (i.e. all of the method types) mandated for public sector provision in South Africa. Injectable contraceptives and male and female condoms were offered at the HIV clinic. Both PHCs offered injectables, oral contraceptive pills (OCPs), male and female condoms, and emergency contraceptive (EC) pills. One PHC offered intrauterine devices (IUDs) via a visiting doctor, and the other PHC offered the implant via an on-site professional nurse. Despite only being provided by one nurse at one site, overall, four respondents (29%) had been trained to insert and remove the implant.

All respondents reported having ever personally used at least one contraceptive method, but only half (7/14, 50%) were currently using a method (Table 1). Three (21%) had had a tubal ligation or hysterectomy, and five (36%) had experience (ever or current) using IUDs (3/14) or implants (2/14). Four (21%) had used EC. The one male participant reported ever and current use of male condoms only. He did not report on contraceptive use by his partner(s).

### Contraceptive counselling and women’s decision making

We asked respondents about what women at the clinics want with regard to contraception. When asked specifically whether women want information on *all* available methods, 79% (11/14) of respondents indicated that, in their experience, women at the clinics do want this information (Table 2).

**Table 2:** Healthcare provider contraceptive beliefs and practice at three public clinics in Johannesburg (n(%))

	HIV clinic (*n* = 5)	PHCs (*n* = 9)	Total (*n* = 14)
Believes women want information on all contraceptive methods	4 (80)	7 (78)	11 (79)
Offers contraceptive counselling to women even if they don’t specifically ask it	5 (100)	8 (89)	13 (93)
Has time to offer counselling/information on all methods to all women who want contraception	4 (80)	8 (89)	12 (86)
Believes his/her clinic meets women’s contraceptive needs	5 (100)	8 (89)	13 (93)

*PHCs* primary healthcare clinics, *SRH* sexual and reproductive health

When asked if they offer contraceptive methods or counselling to women who do not specifically request it, the majority of respondents (13, 93%) indicated they did. As justification, providers expressed concerns about the challenges and risks women face with unplanned pregnancies, including financial implications. Although abortion is legal on demand up to 12 weeks gestation and for several indications through 20 weeks gestation [32], the providers also expressed concerns about health risks if women choose to have an illegal abortion, which is not uncommon in South Africa given poor access to legal services [33], or increased risk of maternal mortality associated with late presentation for antenatal care. Respondents noted that an unplanned pregnancy could also impact on other children in the home. One respondent summed up the reasons for offering contraception to all women:

*Because I believe that we shouldn’t have women who are still falling pregnant while they don’t want to be pregnant. I don’t want people [to] go for backstreet abortion. Maternal mortality -those women die. Women who still have small babies have an impact on the small children. (Medical Officer, HIV Clinic).*

Most respondents indicated that their clinic met patients’ contraceptive needs; however, when asked to elaborate, responses were mixed. Some noted a limited choice of contraceptive methods. Others stressed that they refer women for methods not offered at the clinic. This was most common at the HIV clinic, where the method mix was most limited. Other contraceptive service provision challenges noted across the clinics included stock and staff shortages, staff attitude problems—particularly for young women asking for contraception, and inadequate training on the implant. Perhaps as a result of these challenges, two respondents (1 at the HIV clinic and 1 at the PHCs) indicated that they did not have enough time to offer counselling and information on all contraceptive methods to all women who wanted contraception. Interestingly, these providers had limited personal experience with contraceptive methods and had strong opinions on which methods should and should not be offered to particular types of women.

Prior use of methods by the providers also seemed to influence their method-specific opinions and counselling practices. Providers who had ever used OCPs (9/14, 64%) tended to indicate that this was not a good method for young and HIV-positive women due to forgetfulness and pill burden. Conversely, most providers who indicated that they had personal experience with injectables (11/14, 79%) recommended this method for all types of women. The two providers who had experience using implants were the only two who mentioned possible drug interactions between the method and ARVs. One of the three providers who had used an IUD recommended the method for young women; however, the other two did not. The same two also felt that IUDs were bad for HIV-positive women due to risk of infection. Finally, none of the four providers who used EC recommended this method for any type of woman.

Despite expressing personal opinions that were sometimes contrary, when asked directly, all of the respondents indicated that women should ultimately choose the method that is best for them. Further, when asked how women decide which contraceptive method to choose, respondents indicated that often patients come to the clinic already knowing what method they want, citing reasons of convenience or information obtained from family and friends. Respondents said that the contraceptive methods most often requested by patients were those with the fewest perceived side-effects and those seen to be the most convenient in terms of lifestyle. However, respondents also noted that the women’s information is not always accurate and patient education was needed. Providers also indicated that some women are concerned about the side-effects of certain contraceptive methods, often due to negative past experience, and that women ask questions on which method will best suit them. Finally, most (11/14) respondents expressed that, although women often came to the clinic with a contraceptive method in mind, their final decision about the most appropriate method for them was often based on the information received from the provider. One provider noted:

*Most of the time they don’t want any method that will inconvenience them, they prefer methods with less [side] effects … We educate them because they are coming with the knowledge of what their friends have been using. After giving them education they can be able to choose for themselves. (Enrolled Nurse, PHC1).*

### Which contraceptive methods are recommended (or not) for women

**Fig. 1:**
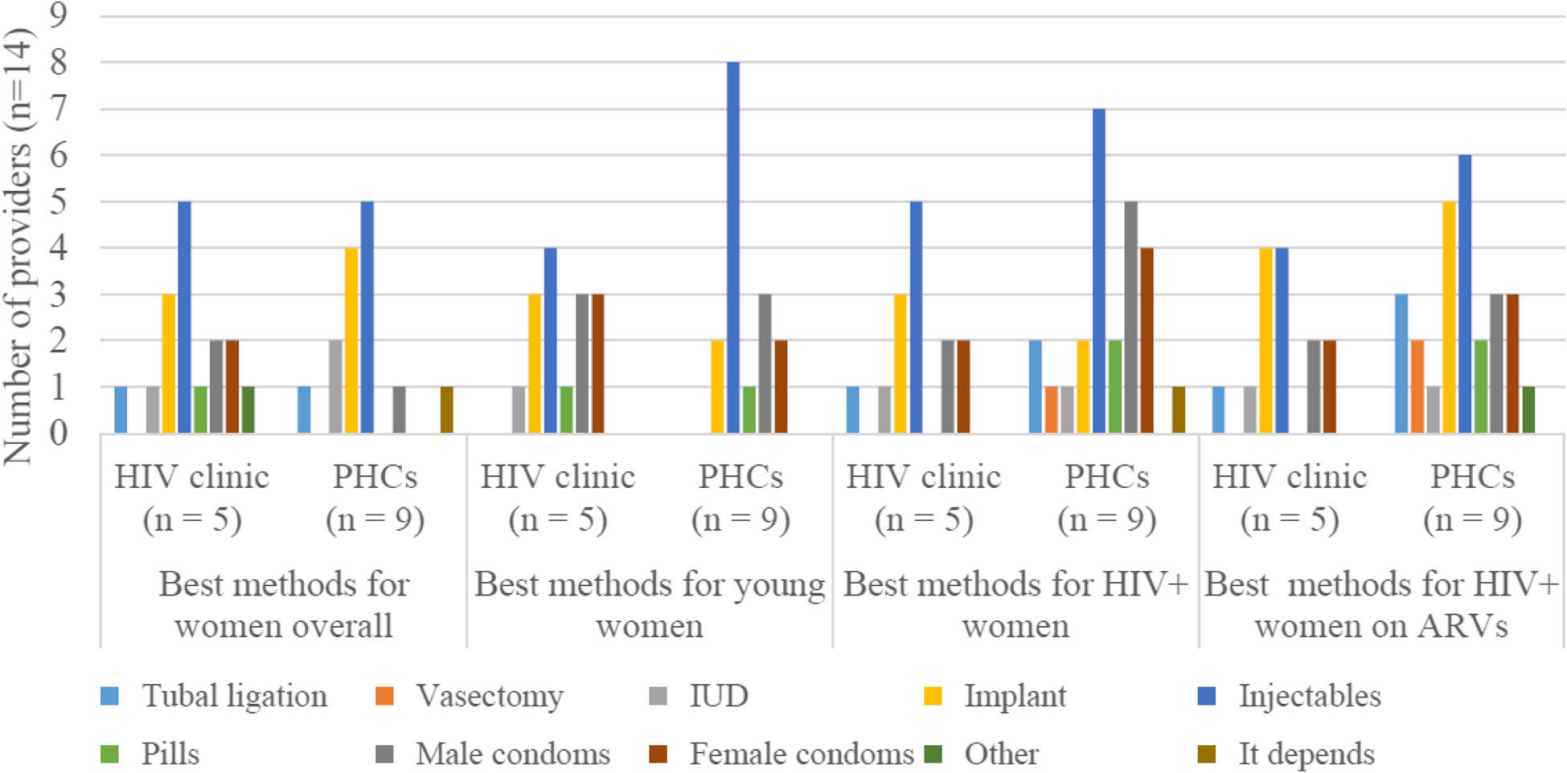
Provider perceptions of “best” contraceptive methods for women, by provider location and type of woman. PHCs = Primary healthcare clinics, ARVs = antiretroviral drugs, IUD = intrauterine contraceptive device

**Fig. 2:**
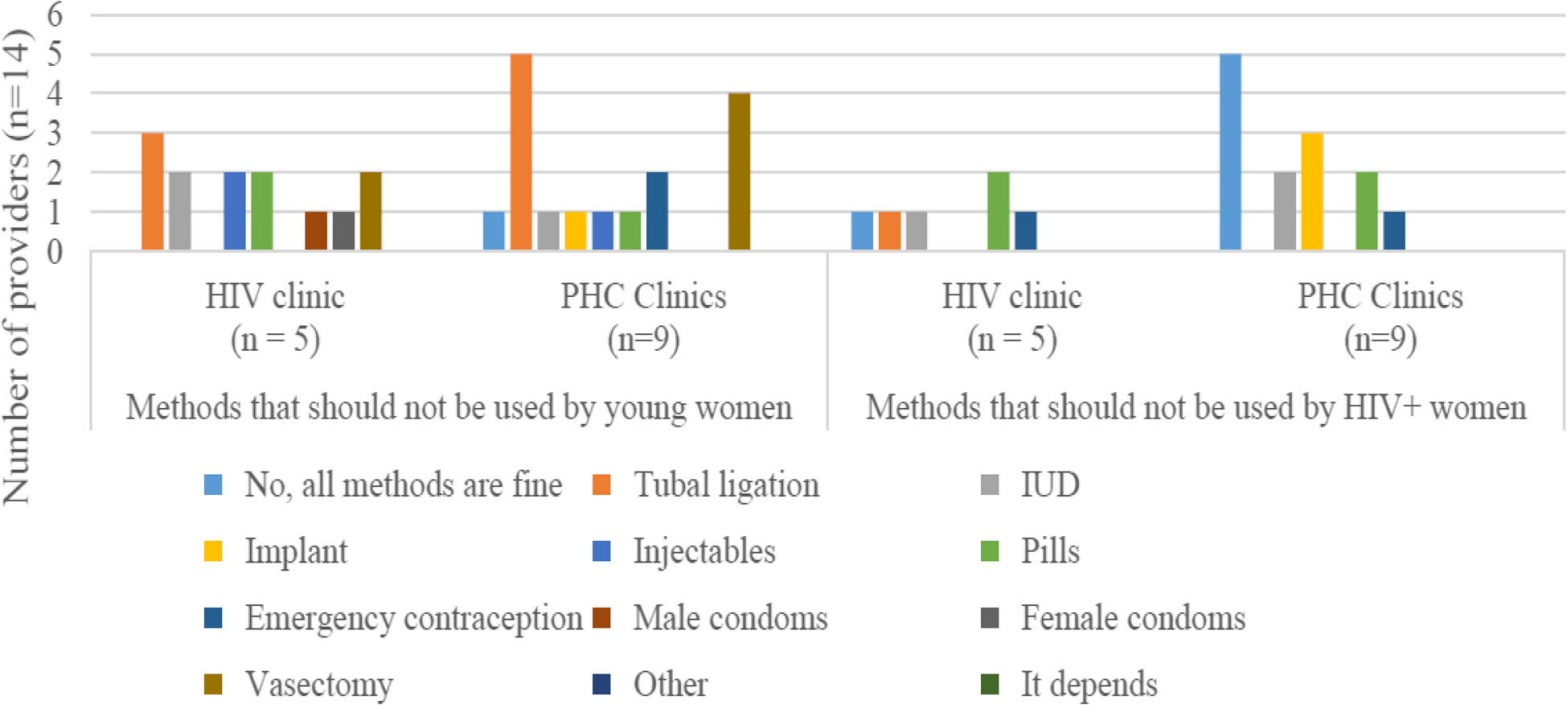
Provider perceptions of contraceptive methods that should not be used, by provider location and type of woman. PHCs = Primary healthcare clinics, ARVs = antiretroviral drugs, IUD = intrauterine contraceptive device

We asked respondents for their opinions on the “best” contraceptive methods for all women in general, and young women and HIV-positive women specifically (Fig. 1). We also asked what makes a good contraceptive method and whether there were any methods that should not be used by young or HIV-positive women, including those taking ARVs (Fig. 2).

Regarding what makes a “good contraceptive method” for all women, almost all (13/14) respondents noted that the method should fit a woman’s needs and lifestyle while also having minimal side-effects:

*It [the contraceptive method] should be acceptable and accessible, and tolerable and reversible. (Medical Officer, HIV Clinic).*

*A method that fits with the woman’s medical and personal lifestyle, a method that a woman is happy about, and has less complications. (Professional Nurse, PHC2).*

Overall, most (10/14) providers (5/5 at the HIV clinic and 5/9 at the PHCs) indicated that injectables were the best methods for women generally as the method has fewer side-effects compared to implants; reduced clinic visits; minimal interaction with other medication, such as ARVs; reduced pill burden; and increased patient compliance.

Half the respondents also recommended implants as a “best method” for all women as it saved time on clinic visits and women could not forget to take it (at least for its effective period). However, there were concerns, with some respondents citing perceived negative side-effects, whether real or anecdotal, including bleeding and ‘risk of infection.’ When asked about use by young and HIV-positive women, opinions about the implant were less favourable. For HIV-positive women in particular, staff had contradictory knowledge and opinions. A third (3/9) of PHC respondents said that HIV-positive women should not use the implant; while at the HIV clinic, no respondent indicated that implant use was precluded for this group. When asked specifically if the implant was a *good* method for women taking ARVs, four HIV clinic staff and five PHC staff members indicated that it was. However, as noted, two providers expressed concerns about interactions with ARVs rendering implants ineffective as a contraceptive method, leading to unwanted pregnancies. One nurse noted that,

*Because of drug interaction to ARVs, most HIV positive women on ARVs fall pregnant while on the implant. (Professional Nurse, PHC2).*

Respondents who felt the implant was not recommended for HIV-positive women indicated that they preferred recommending injectables because these were seen to have fewer side-effects.

*We prefer to give [HIV-positive women] the injectable, because they have less side-effects and drug interaction to ARV’S. (Manager, HIV Clinic).*

Opinions on male and female condoms as contraception were also mixed. Respondents cited the risk of the method bursting, only recommending them for women generally if used as part of a dual protection plan against HIV transmission and pregnancy. However, five of the 14 respondents indicated that male or female condoms were good contraceptive methods for HIV-positive women taking ARVs. In fact, condoms and injectable contraceptives were seen to be the most desirable methods for HIV-positive women due to minimising ‘drug toxicity’ and ‘retransmission.’

Feelings on OCPs were less pronounced with only one respondent noting this as a good method for women generally. Four and three respondents noted that pills should not be used by HIV-positive and young women respectively. When asked to explain, respondents revealed the common local perception that young women were not capable of taking the method correctly.

*Oral contraceptives - they are young and they will fail easily. (Medical Officer, HIV Clinic).*

For HIV-positive women, there was a fear of OCPs contributing to increased pill burden and drug-drug interactions with ARVs. However, for some, the understanding of the drug-drug interactions was limited, as expressed below:

*Oral contraceptives are not good for them [HIV-positive women] at all because it increases the burden of the pill to take. It reduces efficiency on the patients who started on ART because they vomit. (Medical Officer, HIV Clinic).*

Very few respondents indicated that IUDs, tubal ligations, EC and vasectomies were good methods for young or HIV-positive women, with most underscoring that sterilisation was not a good choice for young women as it was ‘too early’ to be used. Just one provider indicated that all methods were appropriate for young women.

Interestingly, one respondent from the HIV clinic spontaneously expressed concern that HIV-positive women ‘abused*’* EC pills, saying that women often came to the clinic for EC indicating that a condom had burst, when in fact there was no actual condom use. The respondent was concerned that HIV-positive women viewed EC as a ‘routine’ form of contraception.

*Most of women coming to this clinic they use emergency contraception as a normal contraception, they don’t use condoms and they always said condoms busted but actually they’re not telling the truth. (Enrolled Nurse, HIV Clinic).*

## Introduction of new contraceptive methods

Two study clinics recently introduced a new contraceptive service. One of the PHCs introduced the implant as part of the national roll-out for PHCs in late 2013 while the HIV clinic began offering injectable contraceptives in April 2014. At the PHC, respondents indicated that no new staff had been hired to manage the introduction of the implant and that all training was done internally with existing staff members. Some PHC respondents felt that introducing the implant offered potential benefits to staff and women, noting that women would not need to return to the clinic for a refill during the method’s effective period, reducing the demand on the nurses who were offering contraceptive services.

*It’s because when [the implant is] inserted, the women will only come back after 3 years for removal and the queues are no longer long for family planning. (Nurse, PHC1).*

However, not all of the PHC respondents agreed that introduction of the implant was wholly beneficial; several reported challenges. Staff selected for implant training missed days of work at the clinic, and respondents noted that this exacerbated existing problems with staff shortages, increasing workloads for those who did not attend. Five respondents at the PHC also noted that ‘many women’ were returning for implant removal before its expiry date complaining of side-effects. Safety of the method was a concern for the respondents, who noted that managing women’s concerns contributed to the overall workload.

Integration of the injectable contraceptive service at the HIV treatment clinic was both similar and different to the implant introduction at the PHC. As the HIV clinic was based within a tertiary hospital, the introduction of the method was subject to hospital-level bureaucracy. In South Africa, contraceptive services are considered to be ‘primary care’ level services, and are not generally offered on an outpatient basis at tertiary hospitals. Thus, according to respondents, the hospital pharmacy reported not having funds in their budget to obtain injectables for distribution in the HIV clinic. Changing this policy required significant intervention by an NGO supporting services at the HIV clinic. Only one of the five HIV clinic respondents noted that introduction of the injectable was beneficial; four indicated that they did not see any benefits. One respondent noted that more education for women is needed to increase awareness of the availability of the service at the clinic.

### Recommendations for contraception scale-up

When asked about the greatest challenges in meeting government goals of increased contraceptive prevalence, respondents highlighted the need to address contraceptive supply, staff shortages and insufficiently trained staff. They also recommended striving to create an environment where women are sufficiently educated to access contraceptive services when they become (more) available. Finally, they felt that supporting current users to prevent discontinuation due to side-effects, misinformation and rumours about methods, and addressing partner issues would be beneficial for contraceptive uptake nationwide.

## Discussion

Our study provides insights on healthcare workers’ experiences personally using and providing contraception at two PHCs and an HIV treatment clinic that offered a limited contraceptive service. Just one respondent was a man, which may actually be over-representative of the gender ratios in nursing in South Africa. Women respondents had personally tried the full range of contraceptive methods available in South Africa’s public sector, and currently 11 of the 14 respondents used a mix of permanent, long- and short-acting methods. The respondents’ personal experience with different methods appeared at times to influence their opinions on which methods were best for different types of women; however, when asked directly, they all indicated that each woman should be free to choose her contraceptive method.

The respondents’ comments on their contraceptive counselling behaviours were both reassuring and questionable. Over half of the respondents indicated that they had received training that included contraceptive counselling and helping women to choose an appropriate method. The respondents acknowledged that women in South Africa need counselling—that women sometimes come to the clinics with misinformation or unaware of existing options. The respondents reported being proactive about offering information on contraceptives, and they expressed that women want information on all available methods. However, they also noted that time constraints and staffing challenges make it difficult to offer full counselling to all women.

Further, some respondent’s opinions about ‘best’ methods for women reflected personal biases, in particular regarding what methods should be made available to young women. According to the respondents, some longer-acting methods, OCPs and emergency contraceptive pills were not appropriate for young women—leaving few choices for this group of individuals. Unfortunately, for young women in South Africa, discriminatory, judgmental attitudes regarding young women’s use of contraceptives have been noted previously elsewhere in the country [25, 34].

Injectable contraceptives have historically dominated the contraceptive method mix in South Africa—for supply- and demand-side reasons [23, 24, 35]. Given this history, it was not surprising that when asked specifically which methods were ‘best’ for women generally, the respondents noted that injectables were best for all women. Implants were also mentioned commonly as a ‘best method’ for women, possibly due to the method’s recent introduction. However, respondents contradicted themselves saying that although the implant was a good method, it had many side-effects and they preferred giving women injectables.

Despite concerns about the quality of the contraceptive counselling and the respondents’ seemingly judgmental attitudes in some cases, it was clear through the interviews that respondents understood women’s needs for contraceptive services, and that poor contraceptive access could result in clinical risks and socioeconomic hardship. Overall, respondents expressed sensitivity to women’s lifestyles and the need to understand a women’s medical history when recommending services.

In 2017, Pleaner et al. documented challenges with rolling out the contraceptive implant in South Africa [36]. Challenges noted by the authors were observed in this study. There was a poor understanding among the respondents of drug-drug interactions and their role in mitigating side-effects. Some respondents seem to have been unaware of the implant’s potential interactions with ARVs, which can adversely affect the implant’s contraceptive efficacy in some women [37]. Side-effects were reportedly the main reason for method discontinuation. This has been reported globally [4]; however, there is also consensus that healthcare providers can help women to mitigate the impact of side-effects by preparing them for short- and long-term effects through counselling. Although the respondents identified side effects as a reason why women might discontinue contraceptive use, they did not express any recognition of their role in potentially helping women to manage those side-effects.

Reflecting on their experiences of adding a new contraceptive method to their service offering, respondents indicated that, despite administrative bureaucracy and staffing challenges around training, the actual introduction did not significantly impact negatively on service delivery. Their bigger concerns were regarding method safety, side-effects, and women’s knowledge of the availability of the method. In fact, ‘demand creation’ was thought to be one of the biggest national challenges with regard to scaling up contraceptive provision. Speaking to this observation for the implant in particular, Pleaner et al. (2017) stressed the importance of managing healthcare providers’ expectations of demand over time – including the “boom bust” phenomenon observed when a new method is introduced and then interest wains [36].

This study has limitations. The respondents represent just three public clinics; however, they had several years of combined service delivery experience in the public sector. We were not able to observe actual service delivery; however, our results are in line with other work on provider attitudes and practice in South Africa, which suggest some areas of quality, but much room for improvement with regard to contraceptive counselling and service provision [5, 25–27].

## Conclusion

Since the 2013 introduction of South Africa’s updated national contraceptive policy, considerable local dialogue about perceived and real progress towards the country’s aim of increasing contraceptive prevalence and reducing unmet need has occurred. There has also been a great deal of debate about the success (or not) of the introduction of the contraceptive implant. This study revealed contradictions in terms of healthcare providers’ beliefs and practice, with respondents at times optimistic about meeting women’s contraceptive needs, but also repeatedly indicating that staff shortages, a lack of training, and a limited contraceptive method mix prevented them from offering integrated, high-quality care. Their comments also reflected misinformation about appropriate usage of some methods.

The pivotal role of the healthcare provider in ensuring access to contraceptive services has been recognized for decades. Providers’ ability to give correct information in an accessible, supportive way is a key component of service quality [6]. Yet, overall, the respondents in this study seemed unaware of their individual potential to improve service delivery. They looked outward – for example, to the potential for more or new staff – to fix current problems. South Africa’s health system is facing significant challenges, and a shortage of human resources is a recognized problem. However, there is no untapped source of new providers waiting to join the ranks and ameliorate current challenges. Further, no additional budgetary allowance is on the horizon. The existing workforce must be capacitated to handle service delivery with the resources available today. This will require creative thinking and training that holistically addresses both healthcare providers’ needs and the unique needs of the women they serve, including young women. Fortunately, there are many resources available on this topic [6, 38–40], which could be adopted into local in-service trainings.

## Supplementary information

**Additional file 1.** Interview guide: Copy of semi-structured guide used for health care provider interviews.

## Data Availability

The data that support the findings of this study are available from the corresponding author but restrictions apply to the availability of these data, which were used with explicit approval for the current study, and so are not publicly available. Data are however available from the authors upon reasonable request and with permission from Human Research Ethics Committee at the University of Witwatersrand (https://www.wits.ac.za/research/researcher-support/research-ethics/ethics-committees/).

## Abbreviations

ART: Antiretroviral Treatment
ARVs: Antiretrovirals
EC: Emergency contraception
HIV: Human Immunodeficiency Virus
IUD: Intrauterine Device
OCP: Oral Contraceptive Pills
PHC: Primary Healthcare Clinics
SRH: Sexual and Reproductive Health
