# Supplementary material for: Provider perspectives on contraceptive service delivery: findings from a qualitative study in Johannesburg, South Africa
Source: BMC Health Serv Res. 2020 Feb 21;20:128. doi: 10.1186/s12913-020-4900-9 (PMC7035764; doi:10.1186/s12913-020-4900-9)
Supplement: Supplementary file 1 — Additional file 1. Interview guide: Copy of semi-structured guide used for health care provider interviews. [file 12913_2020_4900_MOESM1_ESM.pdf]

Today's Date					
d	d	m	m	y	y

Participant ID				

Site ID		

*Instructions to interviewer: Read all questions/text in **bold** out loud. Do not read ALL CAPS text or non-bold response options. Fill out questions 1 and 2 before beginning the interview.*

INTRODUCTION:

Thank you for taking the time to speak with me today. As I have mentioned, for this study, we would like to hear from you about contraceptive services and other sexual and reproductive health services at this clinic. We are particularly interested in your thoughts on provision of contraceptive services to HIV-positive women, including those who might be taking ARVs.

During the interview, we will talk mainly about contraception. "Family planning" is another way we will refer to contraception. There are several methods of contraception. We are interested in all modern methods. Modern methods include sterilization, IUDs (or the loop), the contraceptive implant, injections, pills, emergency contraception, and male and female condoms.

For the purposes of this interview, we will define contraceptive services (or family planning services) as:
- **Counselling on methods,**
- **Provision of a method, and**
- **Removal of a method (if applicable).**

We will also speak about other sexual and reproductive health services, also known as SRH. For this interview, 'SRH services' will include these kinds of services:
- **Sexually Transmitted Infections (STI) services (screening or treatment)**
- **Cervical cancer screening/Pap smears**
- **Breast exams or breast disease management**
- **Pregnancy/delivery**
- **Fertility counselling, care or treatment**
- **Abortion/TOP services**
- **Gender-based violence support services**
- **Services for menopausal women**
- **Services for women with irregular menstruation**

Do you have any questions about what I have described above before we begin?

Today's Date					
d	d	m	m	y	y

Participant ID				

PART A: DEMOGRAPHICS AND PROFESSIONAL EXPERIENCE

Q. #	QUESTION	RESPONSE
**First, I'm going to ask you some brief questions about yourself and your professional experience.**		
**1.**	**I need to indicate your gender. Do you prefer to be identified as [female / male / transgendered]?**	☐ 1 = Male<br>☐ 2 = Female<br>☐ 3 = Other, LIST ______________________
**2.**	**What is your race?**	☐ 1 = Black<br>☐ 2 = White<br>☐ 3 = Colored<br>☐ 4 = Asian<br>☐ 5 = Other, LIST _________________________
**3.**	**When did you finish your education for your job as a** [INSERT JOB TITLE FROM EC]?	____ ____ / ____ ____<br>M M Y Y
**4.**	**How many years of experience do you have working as a** [INSERT JOB TITLE FROM EC]**?**	☐ 1 = < 1 year<br>☐ 2 = ≥ 1 year - < 2 years<br>☐ 3 = ≥ 2 years - < 5 years<br>☐ 4 = ≥ 5 years - < 10 years<br>☐ 5 = ≥ 10 years
**5.**	**I'm going to list some services that you might provide here at this clinic in an average week. For each, I'd like you to tell me how much time you spend providing that kind of service. The options are:**<br><br>1 = **all of your time,**<br>2 = **more than half,**<br>3 = **about half,**<br>4 = **less than half,**<br>5 = **no time.**<br><br>SHOW VISUAL AID.	See table below

Service	Time spent/week
Wellness	
HIV (testing, counselling, ART)	
Ante-natal/maternity	
Family planning	
Other SRH services	
Other non-SRH services	
Management/admin/ meetings	
School health visits	
Other	

Today's Date					
d	d	m	m	y	y

Participant ID				

6.	IF Q5 "OTHER SRH SERVICES" = 5/NO TIME, SKIP TO Q7.<br><br>**You said you provide "other SRH services." Can you tell me if you specifically provide these services:** [LIST RESPONSE OPTIONS].	TICK IF YES<br>☐ 1 = **STI services (screening or treatment)**<br>☐ 2 = **Cervical cancer screening/Pap smears**<br>☐ 3 = **Breast exams or breast disease management**<br>☐ 4 = **Fertility counselling, care or treatment**<br>☐ 5 = **Abortion/TOP services**<br>☐ 6 = **Gender-based violence support services**<br>☐ 7 = **Services for menopausal women**<br>☐ 8 = **Services for women with irregular menstruation**
7.	**How many years of experience do you have with providing contraception to women?**	☐ 0= 0 years, never provided<br>☐ 1 = < 1 year<br>☐ 2 = ≥ 1 year - < 2 years<br>☐ 3 = ≥ 2 years - < 5 years<br>☐ 4 = ≥ 5 years - < 10 years<br>☐ 5 = ≥ 10 years
8.	**a. Were you trained on contraceptive methods as part of your initial training as a** [INSERT JOB TITLE]**?**	☐ 0 = No<br>☐ 1 = Yes
	**b. Have you had any training on contraceptive methods since you became a** [INSERT JOB TITLE]**? This could be in-service training, workshops, etc.**	☐ 0 = No → SKIP TO Q9.<br>☐ 1 = Yes
	bi. IF YES, **How many trainings have you had since your education was completed?**	______________
	c. IF b=YES, **When was the training and what methods were included?** IF MORE THAN THREE, DOCUMENT THE MOST RECENT THREE.	(see table below)

Training 1	Training 2	Training 3
Date	Date	Date
**Methods**<br>☐ 1=Tubal ligation<br>☐ 2=IUD<br>☐ 3=Implant<br>☐ 4=Injectables<br>☐ 5=Pills<br>☐ 6=Emergency contraception<br>☐ 7=Male condoms<br>☐ 8=Female condoms<br>☐ 9=Vasectomy	**Methods**<br>☐ 1=Tubal ligation<br>☐ 2=IUD<br>☐ 3=Implant<br>☐ 4=Injectables<br>☐ 5=Pills<br>☐ 6=Emergency contraception<br>☐ 7=Male condoms<br>☐ 8=Female condoms<br>☐ 9=Vasectomy	**Methods**<br>☐ 1=Tubal ligation<br>☐ 2=IUD<br>☐ 3=Implant<br>☐ 4=Injectables<br>☐ 5=Pills<br>☐ 6=Emergency contraception<br>☐ 7=Male condoms<br>☐ 8=Female condoms<br>☐ 9=Vasectomy

Today's Date					
d	d	m	m	y	y

Participant ID				

		☐ 10=Other, LIST: ____ ☐ 10=Other, LIST: ____ ☐ 10=Other, LIST: ____
	**d. Has any of your contraceptive training included how to help women choose a method that is right for them?**<br><br>di. IF YES, **Please describe that part of the training:**	☐ 0 = No<br>☐ 1 = Yes<br><br>OPEN RESPONSE:
**9.**	**a. In your opinion, do women who come here want information on all available contraceptive methods?**<br><br>**b. Why is that?**<br><br>**c. Do you feel that you have the time to offer information on many contraceptive methods to every woman who wants contraception?**	☐ 0 = No<br>☐ 1 = Yes<br><br>OPEN RESPONSE:<br><br>☐ 0 = No<br>☐ 1 = Yes
**10.**	**a. Have you been trained on insertion of the implant?**<br><br>**b.** IF a=YES, **Have you provided implants to women?**	☐ 0 = No → SKIP TO 10D.<br>☐ 0 = Yes<br><br>☐ 0 = No<br>☐ 1 = Yes → SKIP TO 10D.

Today's Date					
d	d	m	m	y	y

Participant ID				

	**c.** IF b=NO, **Why not?**	OPEN RESPONSE:
	**d. Have you been trained on removal of the implant?**	☐ 0 = No → SKIP TO Q11.<br>☐ 1 = Yes
	**e. Have you removed any implants?**	☐ 0 = No<br>☐ 1 = Yes → SKIP TO Q10G.
	**f.** IF e=NO, **Why not?**	OPEN RESPONSE:
	g. IF e=YES, **Please describe your experience with removing the implant**	OPEN RESPONSE:

Today's Date					
d	d	m	m	y	y

Participant ID				

**11.**	**a. Do you currently practice at another location in addition to this one?**	☐ 0 = No → SKIP TO Q12.<br>☐ 1 = Yes
	b. IF a=YES, **What type of facility is the other location?**	☐ 1 = Public hospital<br>☐ 2 = Public clinic<br>☐ 3 = Private hospital<br>☐ 4 = Private clinic<br>☐ 5 = Own practice<br>☐ 6 = Other, LIST: _______________
	c. IF a=YES, **Do you personally provide contraception there?** IF YES, **what methods do you provide?**	☐ 0 = No → SKIP TO 11D.<br>☐ 1 = Yes, LIST:<br>☐ 1 = Tubal ligation<br>☐ 2 = IUD<br>☐ 3 = Implant<br>☐ 4 = Injectables<br>☐ 5 = Pills<br>☐ 6 = Emergency contraception<br>☐ 7 = Male condoms<br>☐ 8 = Female condoms<br>☐ 9 = Other, LIST: _______________
	d. IF a=YES, **Do you provide other SRH services there? If so, please describe what you do.**	☐ 0 = No → SKIP TO Q12.<br>☐ 1 = Yes, LIST:<br>☐ 1 = Ante-natal care<br>☐ 2 = Maternity services<br>☐ 3 = STI services (screening or treatment)<br>☐ 4 = Cervical cancer screening/Pap smears<br>☐ 5 = Breast exams or breast disease management<br>☐ 6 = Fertility counselling, care or treatment<br>☐ 7 = Abortion/TOP services<br>☐ 8 = Gender-based violence support services<br>☐ 9 = Services for menopausal women<br>☐ 10 = Services for women with irregular menstruation

Today's Date					
d	d	m	m	y	y

Participant ID				

**12.**	**a. I'd like to know about your own experiences with contraception. Can you tell me if you or your partner have <u>ever</u> used any of these methods for contraception:**	☐ 0 = Never used a method (SKIP TO Q13).<br><br>Has used:<br>☐ 1 = **Tubal ligation**<br>☐ 2 = **IUD**<br>☐ 3 = **Contraceptive Implant**<br>☐ 4 = **Injectables**<br>☐ 5 = **Pills**<br>☐ 6 = **Emergency contraception**<br>☐ 7 = **Male condoms**<br>☐ 8 = **Female condoms**<br>☐ 9 = **Male vasectomy**<br>☐ 10 = **Other**, LIST: _______________
	**b.** IF S/HE HAS EVER USED ANY METHOD, **And would you mind telling me if you or your partner currently used any of these methods for contraception:**	☐ 0 = Not applicable<br><br>Currently uses:<br>☐ 1 = **Tubal ligation**<br>☐ 2 = **IUD**<br>☐ 3 = **Contraceptive Implant**<br>☐ 4 = **Injectables**<br>☐ 5 = **Pills**<br>☐ 6 = **Emergency contraception**<br>☐ 7 = **Male condoms**<br>☐ 8 = **Female condoms**<br>☐ 9 = **Male vasectomy**<br>☐ 10 = **Other**, LIST: _______________

Today's Date					
d	d	m	m	y	y

Participant ID				

PART B: WOMEN'S CONTRACEPTIVE NEEDS AND ACCESS AT THIS CLINIC

Q. #	QUESTION	RESPONSE
**Now I'm going to ask you a few questions about women's family planning needs and access when they come to this clinic.**		
**13.**	**a. Thinking about the women you see here at this clinic, what contraceptive methods do you think are best for them?** [MARK ALL THAT APPLY.]	☐ 1 = Tubal ligation<br>☐ 2 = IUD<br>☐ 3 = Implant<br>☐ 4 = Injectables<br>☐ 5 = Pills<br>☐ 6 = Emergency contraception<br>☐ 7 = Male condoms<br>☐ 8 = Female condoms<br>☐ 9 = Other, LIST: _______________<br>☐ 10 = None<br>☐ 11 = It depends
	**b. Why do you think that?**	OPEN RESPONSE:

Today's Date					
d	d	m	m	y	y

Participant ID				

**14.**	**a. What about contraceptive methods for young women? What methods are best for them?** [MARK ALL THAT APPLY.]	☐ 1 = Tubal ligation<br>☐ 2 = IUD<br>☐ 3 = Implant<br>☐ 4 = Injectables<br>☐ 5 = Pills<br>☐ 6 = Emergency contraception<br>☐ 7 = Male condoms<br>☐ 8 = Female condoms<br>☐ 9 = Vasectomy<br>☐ 10 = Other, LIST: _______________<br>☐ 11 = None, they shouldn't use any methods<br>☐ 12 = It depends
	**b. Why do you think that?**	OPEN RESPONSE:
	**c. Are there any methods that you think young women should NOT use?**	☐ 0 = No, All methods are fine for young women<br><br>Young women should NOT use:<br>☐ 1 = Tubal ligation<br>☐ 2 = IUD<br>☐ 3 = Implant<br>☐ 4 = Injectables<br>☐ 5 = Pills<br>☐ 6 = Emergency contraception<br>☐ 7 = Male condoms<br>☐ 8 = Female condoms<br>☐ 9 = Vasectomy<br>☐ 10 = Other, LIST: _______________<br>☐ 11 = It depends

Today's Date					
d	d	m	m	y	y

Participant ID				

	**d. Why do you think that?**	OPEN RESPONSE:
**15.**	**a. What about contraceptive methods for HIV-positive women? What methods are best for them?** [MARK ALL THAT APPLY.]	☐ 1 = Tubal ligation<br>☐ 2 = IUD<br>☐ 3 = Implant<br>☐ 4 = Injectables<br>☐ 5 = Pills<br>☐ 6 = Emergency contraception<br>☐ 7 = Male condoms<br>☐ 8 = Female condoms<br>☐ 9 = Vasectomy<br>☐ 10 = Other, LIST: _______________<br>☐ 11 = It depends
	**b. Why do you think that?**	OPEN RESPONSE:

Today's Date					
d	d	m	m	y	y

Participant ID				

	**c. Are there any methods that you think HIV-positive women should NOT use?**	☐ 0 = No, All methods are fine for HIV-positive women<br><br>HIV-positive women should NOT use:<br>☐ 1 = Tubal ligation<br>☐ 2 = IUD<br>☐ 3 = Implant<br>☐ 4 = Injectables<br>☐ 5 = Pills<br>☐ 6 = Emergency contraception<br>☐ 7 = Male condoms<br>☐ 8 = Female condoms<br>☐ 9 = Vasectomy<br>☐ 10 = Other, LIST: _______________<br>☐ 11 = It depends
	**d. Why do you think that?**	OPEN RESPONSE:
**16.**	**a. What methods do you think can be used by women who are HIV-positive and taking ARVs?** [MARK ALL THAT APPLY]	☐ 0 = All methods are fine for HIV-positive women taking ARVs<br>☐ 1 = Tubal ligation<br>☐ 2 = IUD<br>☐ 3 = Implant<br>☐ 4 = Injectables<br>☐ 5 = Pills<br>☐ 6 = Emergency contraception<br>☐ 7 = Male condoms<br>☐ 8 = Female condoms<br>☐ 9 = Vasectomy<br>☐ 10 = Other, LIST: _______________<br>☐ 11 = It depends

Today's Date					
d	d	m	m	y	y

Participant ID				

	b. **Do you think that the implant is a good choice of method for women who are taking ARVs?**<br><br>**c. Why do you think that?**	☐ 0 = No<br>☐ 1 = Yes<br><br>OPEN RESPONSE:
**17.**	**What do you think makes a contraceptive method a good method for a particular woman?**	OPEN RESPONSE:
**18.**	**How do you think women decide what contraceptive method to ask for or take at a health facility?**	OPEN RESPONSE:

Today's Date					
d	d	m	m	y	y

Participant ID				

**19.**	**Sometimes women stop using a contraceptive method. Given your experience offering contraception, what do you think are the top three reasons why that happens?** [READ ALL OPTIONS OUT LOUD. MARK THREE.]	☐ 0= N/A = Respondent doesn't have experience offering contraception<br><br>☐ 1 = **Side effects**<br>☐ 2 = **Rumors or misinformation about contraception**<br>☐ 3 = **Desire for pregnancy**<br>☐ 4 = **Lack of understanding of the need to renew on time**<br>☐ 5 = **Partner issues (e.g. new partner/ partner wants her to stop, etc.)**<br>☐ 6 = **Family/community pressure to not use contraception**<br>☐ 7 = **Can't get the method she wants**<br>☐ 8 = **Other:** ____________________________
**20.**	**Do you ever offer methods or counselling on contraception to women even if they don't specifically ask for contraception during their visit?**<br><br>**c. Why is that?**	☐ 0 = No<br>☐ 1 = Yes<br><br>OPEN RESPONSE:
**21.**	**a. Do you think men should be more involved in contraceptive service provision and use?**<br><br>**b. Why do you think that?**	☐ 0 = No<br>☐ 1 = Yes<br>☐ 2 = Don't know/not sure<br><br>OPEN RESPONSE:

Today's Date					
d	d	m	m	y	y

Participant ID				

	**c.** IF a=YES, **How do you think men could be encouraged to get more involved in contraception provision or uptake?**	OPEN RESPONSE:
**22.**	**Some people believe that there are too many teenage pregnancies in South Africa. What do you think could be done to encourage or help more young women to come for contraception?**	OPEN RESPONSE:
**23.**	**a. In your opinion, is this clinic meeting the needs of the women who come here with regard to contraception services?**	☐ 0 = No<br>☐ 1 = Yes<br>☐ 2 = Don't know/not sure
	**b. Can you please explain why you think that?**	OPEN RESPONSE:

Today's Date					
d	d	m	m	y	y

Participant ID				

**24.**	**a. Do you think there are any challenges at this clinic with regard to contraception service provision?**<br><br>**b. Can you please explain why you think that?** [ASK HIM/HER TO LIST THE CHALLENGES IF a = YES.]	☐ 0 = No<br>☐ 1 = Yes<br>☐ 2 = Don't know/not sure<br><br>OPEN RESPONSE:
**25.**	**Is there anything that you would recommend for improving contraceptive service provision at this clinic?**<br><br>**b. Can you please explain why you think that?**	☐ 0 = No<br>☐ 1 = Yes<br>☐ 2 = Don't know/not sure<br><br>OPEN RESPONSE:

Today's Date					
d	d	m	m	y	y

Participant ID				

**26.**	**a. Do you know if the clinic has any targets for increasing contraceptive service provision – for certain methods or all methods?**	☐ 0 = No → SKIP TO Q27.<br>☐ 1 = Yes<br>☐ 2 = Don't know/not sure → SKIP TO Q27.
	**b. What are the targets?**	OPEN RESPONSE:
	**c. Do you think working towards those targets will have an impact on your work at the clinic?**	☐ 0 = No → SKIP TO Q27.<br>☐ 1 = Yes<br>☐ 2 = Don't know/not sure → SKIP TO Q27.
	**d. In what way?**	OPEN RESPONSE:
**27.**	**The National Department of Health has some goals for increasing contraceptive uptake in South Africa. In your opinion, what do you think the biggest challenges would be in scaling up the contraceptive services at this clinic?**	OPEN RESPONSE:

Today's Date					
d	d	m	m	y	y

Participant ID				

**28.**	**a. The National Department of Health also has goals for making contraceptive services an integrated part of health services provision. What does it mean to you for a service to be "integrated"?**	OPEN RESPONSE:
	**b. Do you think the contraceptive service here is "integrated?"**	☐ 0 = No<br>☐ 1 = Yes → SKIP TO Q29.<br>☐ 2 = Don't know/not sure
	**c. What would be needed to make contraceptive services at this facility an integrated part of the care that is offered?**	OPEN RESPONSE:

Today's Date					
d	d	m	m	y	y

Participant ID				

PART C: WOMEN'S SRH NEEDS AT THIS CLINIC

Q. #	QUESTION	RESPONSE
**Now I would like to ask you about some of the other SRH services that may be available at this clinic.**		
**29.**	**In your opinion, what other kinds of sexual and reproductive health, or SRH, services do the women who come to this clinic need?** [PROBE FOR ANY THAT ARE NOT MENTIONED.]	☐ 1 = None<br>☐ 2 = Ante-natal care<br>☐ 3 = Maternity services<br>☐ 4 = STI services (screening or treatment)<br>☐ 5 = Cervical cancer screening/Pap smears<br>☐ 6 = Breast exams or breast disease management<br>☐ 7 = Fertility counselling, care or treatment<br>☐ 8 = Abortion/TOP services<br>☐ 9 = Gender-based violence support services<br>☐ 10 = Services for menopausal women<br>☐ 11 = Services for women with irregular menstruation<br>☐ 12 = Other ______________________
**30.**	**For each of these SRH services, can you tell me if the service is offered here or referred out to another facility?**	

	Offered here = 1, Referred = 2	If referred, where to:
Ante-natal care		
Maternity services		
STI services (screening or treatment)		
Cervical cancer screening/Pap smears		
Breast exams or breast disease management		
Fertility counselling, care or treatment		
Abortion/TOP services		
Gender-based violence support services		
Services for menopausal women		
Services for women with irregular menstruation		
Other ____________________		

Today's Date					
d	d	m	m	y	y

Participant ID				

**31.**	**a. In your opinion, is this clinic meeting the needs of women with regard to SRH services?**<br><br>**b. Can you please explain why you think that?**	☐ 0 = No<br>☐ 1 = Yes<br>☐ 2 = Don't know/not sure<br><br>OPEN RESPONSE
**32.**	**a. Do you think there are any challenges at this clinic with regard to SRH service provision?**<br><br>**b. Can you please explain why you think that?** [ASK HIM/HER TO LIST THE CHALLENGES IF a = YES.]	☐ 0 = No<br>☐ 1 = Yes<br>☐ 2 = Don't know/not sure<br><br>OPEN RESPONSE

Today's Date					
d	d	m	m	y	y

Participant ID				

**33.**	**a. Is there anything that you would recommend for improving SRH service provision at this clinic?**	☐ 0 = No<br>☐ 1 = Yes<br>☐ 2 = Don't know/not sure
	**b. Can you please explain why you think that?**	OPEN RESPONSE
**34.**	**The National Department of Health also has some goals for making SRH services an integrated part of health services provision.**	
	**a. Do you think the SRH services here are "integrated?"**	☐ 0 = No<br>☐ 1 = Yes → SKIP TO Q35.<br>☐ 2 = Don't know/not sure
	**b. In your opinion, what do you think would be needed to make SRH service provision an integrated part of the care that is offered here?**	OPEN RESPONSE

Today's Date					
d	d	m	m	y	y

Participant ID				

**35.**	**I'd like to ask specifically about abortion, or TOP, services. Many women struggle to find a legal/safe provider in South Africa, and many go to backstreet providers.**	
	**a. Do you think TOP services should be available in every public facility that offers contraceptive services?**	☐ 0 = No<br>☐ 1 = Yes → SKIP TO Q36.<br>☐ 2 = Don't know/not sure
	**b. Can you say why you think that?**	OPEN RESPONSE

Today's Date					
d	d	m	m	y	y

Participant ID				

PART D: INTRODUCTING CONTRACEPTION /NEW CONTRACEPTIVE METHODS

Q. #	QUESTION	RESPONSE
**We understand that [the implant / contraception] was introduced at this clinic. We would like to talk with you about your thoughts on the benefits and challenges of introducing this new service here.**<br><br>NB: USE "IMPLANT" AT THE PHCS AND "CONTRACEPTION" AT THE HOSPITAL-BASED HIV CARE AND TREATMENT CLINIC.		
**36.**	**a. Did the clinic have to hire or find new staff to manage the [implant / contraceptive] service?**<br><br>**b. Why or why not?**	☐ 0 = No<br>☐ 1 = Yes<br>☐ 2 = Don't know/not sure<br><br>OPEN RESPONSE
**37.**	**a. Did the clinic have to find extra money, or add money to its budget, in order to make [the implant / contraceptives] available here?**<br><br>**b. Why or why not?**	☐ 0 = No<br>☐ 1 = Yes<br>☐ 2 = Don't know/not sure<br><br>OPEN RESPONSE

Today's Date					
d	d	m	m	y	y

Participant ID				

**38.**	**What were some of the challenges related to introduction of [the implant / a contraceptive service] here?**	OPEN RESPONSE
**39.**	**What were some of the benefits related to introduction of [the implant / a contraceptive service] here?**	OPEN RESPONSE
**40.**	**What recommendations do you have for other [primary clinics / HIV care and treatment sites] that might be considering introducing [implant / contraceptive] services?**	OPEN RESPONSE

Today's Date					
d	d	m	m	y	y

Participant ID				

**41.**	**We have finished the interview. Thanks for your time. Do you have any questions for me?**	☐ 0 = No<br>☐ 1 = Yes<br><br>LIST IF YES:

**Interviewer initials and date:**	**Comments:**
